# Discovery of age-related early-stage glycated proteins based on deep quantitative serum glycated proteome analysis: Age-related changes in the early-glycated proteome

**DOI:** 10.3724/abbs.2023222

**Published:** 2023-08-31

**Authors:** Xinyue Ren, Linlin Wu, Lei Zhang, Yang Liu, Guoli Wang, Haojie Lu

**Affiliations:** 1 Shanghai Medical College Fudan University Shanghai 200032 China; 2 Institutes of Biomedical Sciences and Department of Chemistry and NHC Key Laboratory of Glycoconjugates Research Fudan University Shanghai 200032 China

**Keywords:** aging, early glycation, glycated proteome

## Abstract

Aging is a pressing global health issue that is linked to various diseases, such as diabetes and Alzheimer’s disease. It is well known that glycation plays a pathological role in the aging process and age-related diseases. Thus, it is of great significance to discover protein glycation at an early stage for monitoring and intervention in the aging process. However, the endogenous age-related early-stage glycated proteome remains insufficiently profiled. To address this research gap, our study focuses on assessing glycated proteomics profiles in the serum of mice. We employ a robust and quantitative strategy previously developed by our team, to analyze endogenous glycated proteome in serum samples of 4 age groups of mice (10 weeks, 16 weeks, 48 weeks and 80 weeks). In total, 2959 endogenous glycated peptides corresponding to 296 serum proteins are identified from 48 runs of serum samples from 16 mice across the four age groups. By comparing these glycated peptides between adjacent age groups, we discover 49 glycated peptides from 35 proteins that show significant upregulation between the 48-week and 80-week age groups. Furthermore, we identify 10 glycated proteins (or protein groups) that are significantly upregulated only between the 48-week and 80-week age groups, including lecithin-cholesterol acyltransferase (LCAT) and apolipoprotein A-II (Apo A-II). These novel findings provide unique signatures for understanding the aging process and age-related diseases. By shedding light on the early-stage glycated proteome, our study contributes valuable insights that may have implications for future interventions and therapeutic approaches.

## Introduction

The global population of individuals aged 65 years or older is rapidly increasing and is projected to reach 1.5 billion by 2050 [1]. This age group is particularly susceptible to chronic diseases, accounting for a significant proportion of deaths [ 2, 3]. Consequently, aging stands as a primary risk factor for morbidity and mortality in modern society. To better understand the aging process, predict accelerated health decline, and develop interventions for age-related diseases, the identification of serum signatures that systematically change with age is of paramount importance [ 4, 5]. Proteins play a crucial role in physiological and pathological functions, making them a valuable source for discovering molecular signatures of chronological and biological age in serum. With advancements in proteomic platforms, the assessment of thousands of proteins from biological samples has led to the discovery of numerous aging-related proteomic signatures [6]. Proteomic biomarkers hold promise for diagnosing age-related diseases and evaluating the effectiveness of interventions [ 7 , 8]. However, focusing solely on protein concentration changes may not fully reveal the molecular basis underlying the aging mechanism, as the function of proteins can be significantly affected by their post-translational modifications.

One such post-translational modification is glycation, which may impair protein function and characteristics [8]. Early-stage glycation proteins are formed through the nonenzymatic covalent attachment of reducing sugars or sugar derivatives ( *e.g.*, glucose, fructose) to proteins [9], and they can progress to the formation of advanced glycation end-products (AGEs) through various reactions [10]. It has been shown that the glycation of proteins is closely associated with aging and age-related diseases [ 11– 17]. For instance, the level of AGEs was found to increase significantly with old age [18], and the accumulation of AGEs is involved in exacerbating the development of complications of diabetes and increased risk of cardiovascular diseases and premature death [19]. Therefore, the identification of age-related glycated proteins may provide new clues to effectively monitor and manage the aging process and to explore the mechanisms underlying the relationship between aging and aging-related diseases.

Detecting early-stage glycation is vital for early monitoring and better intervention in aging-related deterioration [10]. Some well-known early-stage glycated markers, such as glycated hemoglobin and glycated albumin, have been utilized as indicators for blood glucose control monitoring, while early-stage glycated transferrin shows potential for monitoring blood glucose control and early diagnosis of diabetic nephropathy [ 15, 20, 21]. These findings underscore the importance of studying early-stage glycated proteins in biological processes. However, current research on early-stage glycated proteins associated with aging deterioration primarily focuses on a limited number of high-abundance proteins, leaving the extent of glycation modifications in many other serum proteins unclear. Comprehensive profiling of the glycated proteome in complex biological samples has been challenging due to the labile nature of glycation and ionization suppression of low-abundance proteins or peptides by high-abundance counterparts. Recent advancements in analytical methods have provided opportunities to overcome these challenges, enabling in-depth, high-throughput, and highly reproducible analysis of endogenous glycated proteins [22]. In this study, we employed this method based on filter-assisted sample preparation to comprehensively profile the endogenous age-related early-stage glycated proteome.

Mice serve as an excellent model system to investigate human aging [23], making the analysis of the serum early-stage glycated proteome during mouse aging a valuable approach to understand the relationship between protein glycation and aging. However, studies on the dynamic changes in glycated proteins during the aging process in mice are lacking. Therefore, this study systematically investigated the changes in serum early-stage protein glycation from age 10 weeks to age 80 weeks at four time points in mice. Our analysis identified a total of 2959 endogenous glycated peptides corresponding to 296 serum proteins from four different age groups. By comparing these glycated peptides between adjacent age groups, we identified 10 glycated proteins (or protein groups) that were significantly upregulated only between the 48-week and 80-week age groups. These findings shed light on novel glycated proteins and their potential involvement in the aging process, offering valuable aging biomarker candidates and new insights into the molecular mechanisms underlying aging.

## Materials and Methods

### Materials and chemicals

Acetonitrile was obtained from Merck (Darmstadt, Germany), while N-glycosidase F (PNGase F, 500 U/μL) was obtained from New England Biolabs (Ipswich, USA). Trypsin was sourced from Beijing Shengxia Proteins Scientific Ltd. (Beijing, China). The bicinchoninic acid protein assay kit and HeLa protein digest standard were acquired from Pierce (Rockford, USA). Unless specified otherwise, all other chemical reagents were obtained from Sigma-Aldrich (St Louis, USA). Distilled water was purified using a Milli-Q system (Millipore, Milford, USA). Ultrafiltration tubes (MWCO=10 kDa) were purchased from Millipore (Amicon Ultra; Merck), and MonoSpin C18 was obtained from Shimadzu (Kyoto, Japan). Affi-Gel boronate gel was sourced from Bio-Rad Laboratories (Hercules, USA).

### Mouse samples

Male C57BL/6 mice, aged four weeks, were procured from the Shanghai Laboratory Animal Center of the Chinese Academy of Sciences (Shanghai, China). The mice were housed under a 12-hour light/dark cycle at constant temperature and humidity with access to food and water ad libitum. Blood samples were collected using retro-orbital bleeding at 10 weeks, 16 weeks, 48 weeks, and 80 weeks, with four mice at each time point, using pro-coagulation tubes. Serum samples were obtained by centrifuging the collected blood at 3000 *g* for 10 min and stored at −80°C for subsequent analysis. The Institutional Animal Care and Use Committee of Fudan University approved all animal protocols.

### Filter-aided sample preparation

The filter-aided sample preparation was performed using the method we previously developed [22]. First, the serum samples were reduced with sodium cyanoborohydride (NaBH3CN) in 1× phosphate-buffered saline individually for 4 h at 37°C. Afterwards, the reductant NaBH3CN was removed by Amicon ultrafiltration while using ammonium bicarbonate (ABC) as washing buffer each time. The proteins were then reduced with dithiothreitol (DTT) followed by iodoacetamide (IAA) at room temperature in the dark. After removal of DTT and IAA by ultrafiltration, one unit of PNGase F solution was added and incubated at 37°C overnight to remove N-glycans from proteins. The samples were then purified with ABC buffer. After that, the proteins were recovered using reverse centrifugation. Subsequently, protein quantification was performed by ultraviolet spectrophotometry, with 500 μg of protein reserved in each tube for further analysis.

### Protein digestion and selective enrichment of glycated peptides

The serum proteins prepared through FASP were denatured in a 100°C water bath for 6 min and then cooled to room temperature. Proteins in 50 mM ABC solution (one microgram per microliter) were digested overnight at 37°C at a final trypsin-to-protein ratio of 1:50 (w/w). The reaction mixtures were again bathed at 100°C to terminate protease digestion. Subsequently, glycated peptides were enriched from the mixtures using Affi-Gel boronate gel for 16 h at 37°C and 1100 rpm according to the method we previously developed [22]. The collected peptides were purified using MonoSpin C18, lyophilized to dryness, and stored at −80°C until further analysis.

### Nano-high-performance liquid chromatography−MS/MS profiling of glycated peptides

The glycated peptides were reconstituted with 10 μL of solvent A (2% acetonitrile, 0.1% formic acid, and 98% water) and separated on a reversed-phase chromatography column (PepMap C18, 1.6 μm, 250 mm×75 μm; Ionopticks, Melbourne, Australia). Online analysis was performed with a quadrupole time-of-flight mass spectrometer (timsTOF Pro; Bruker Daltonics, Billerica, USA). The LC-MS parameters were as follows. The column flow rate was maintained at 300 nL/min with a gradient of solvent B (98% acetonitrile, 0.1% formic acid, and 2% water), from 2% to 22% over 45 min, followed by 22% to 37% over 5 min, from 37% to 80% in the subsequent 5 min, with an 80% hold for 5 min. Finally, the chromatography column was re-equilibrated with the initial conditions for 2 min. Full-scan mass spectra were acquired across the mass range of m/z 100–1700 with an accumulation and ramp time of 100 ms. The single cycle acquisition period was set as 1.16 sec, which included one full TIMS MS scan and 10 parallel accumulation serial fragmentation (PASEF) MS/MS scans. During the PASEF MS/MS scans, the collision energy increased linearly as a function of mobility. The intensity threshold was set at 5000; the range of 1/k0 was 0.7–1.3 Vs/cm ^2^; the capillary voltage was 1500 V; the auxiliary gas flow rate was 3 L/min; the ionization temperature was 180°C; and the column temperature was 50°C.

The HeLa protein digest standard was selected as the standardized reference sample to evaluate the LC-MS system performance for label-free quantification using the LC-MS/MS method described above.

### Database search and label-free quantification of glycated peptides

We searched all the acquired data files using PEAKS Online against the mouse database (downloaded in Feb 2021; Mus musculus, 17063 entries). Enzyme specificity was set as trypsin with up to four missed cleavages. Searches were performed with carbamidomethyl cysteine (+57.021 Da) as a fixed modification and methionine oxidation (+15.995 Da), protein N-term acetylation (+42.010 Da), lysine glycation (+164.068 Da), and asparagine deamidation (+0.984 Da) as variable modifications.The false discovery rate (FDR) was set at 1%. The precursor mass error tolerance was set at 20 ppm, and the fragment mass error tolerance was set at 0.05 Da. The identified peptides within 6 or beyond 45 amino acids were excluded.

For label-free relative quantification analysis, MS1 signal intensity was extracted, and a standard PEAKS Online analysis strategy (PEAKSQ) that used an aggregate of all MS1 peptide intensities of a reported protein was followed. Moreover, the match-between-runs algorithm embedded in PEAKS Online was applied for peptide identification across runs based on high-quality mass accuracy and normalized retention times.

### Data processing for bioinformatics analysis

Further processing of the data from PEAKS Online analysis was performed to obtain volcano plots, which are accessible on the online website https://www.metaboanalyst.ca/. Normalization by median and a non-parametric test were used for the data processing to make volcano plots. The amino acid sequence analysis before and after the glycation site of the peptide was analyzed by the online website https://weblogo.berkeley.edu/logo.cgi. The Gene Ontology was produced by the website https://david.ncifcrf.gov/. The Pearson correlation coefficients, clusters of glycated proteins and abundance distribution of glycated proteins were produced by the R packages.

## Results

### Quantitative profiling of serum-glycated peptides in different age groups of mice

**Figure 1:**
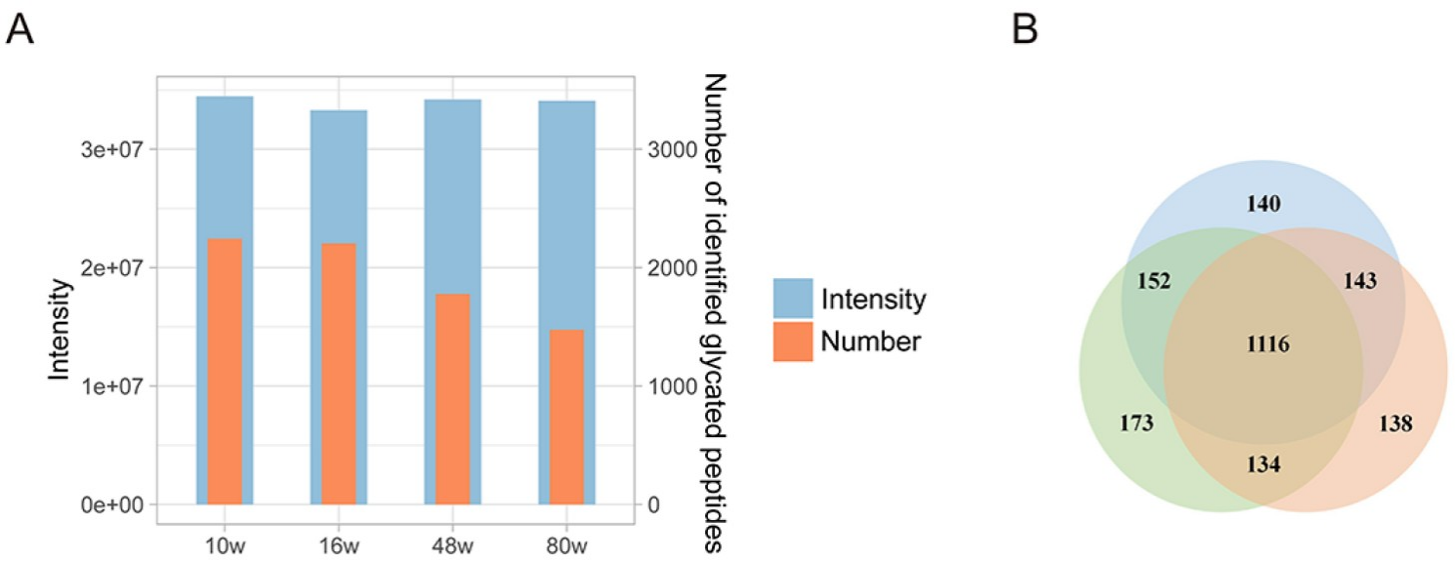
Figure 1 Identification scale and replication (A) Overview of the number of identified glycated peptides in label-free quantification. Each orange column represents the number of identified glycated peptides in an age group. Each blue column represents the average value of 12 runs at each age group after summing up the signal intensity of all glycated peptides in each run. (B) The overlap diagram of the identified glycated peptides in three technical replicates of sample 5 shown by Venn diagram. Each circle represents the number of identified glycated peptides in a single run.

In this study, we identified a total of 2959 glycated peptides, representing the largest scale of mouse serum glycated peptide identification to our knowledge. Among the different age groups (10 w, 16 w, 48 w, and 80 w), we detected 2244, 2205, 1777, and 1474 glycated peptides, respectively ( Figure 1A, orange columns). To ensure the reliability of our identification results, we further analyzed the total signal intensity of each group and found that the total signal intensity was comparable for all age groups ( Figure 1A, blue columns). Additionally, by taking sample 5 as an example, we observed a significant overlap of identified glycated peptides among technical replicates, with 77.4% of glycated peptides quantified in at least two replicates and 55.9% quantified in all three replicates ( Figure 1B), indicating excellent experimental reproducibility.

### Evaluation of the performance of label-free quantitation of early glycated proteins

To ensure the reliability of our quantitative results, we employed the FASP approach to enhance the reproducibility of sample preparation. Additionally, we included standardized reference samples (HeLa protein digest standards) in the analysis of serum samples under the same LC-MS conditions. The average Pearson correlation coefficient of all identified glycated peptides in the serum samples was 0.69 ( Supplementary Figure S1A), while the average correlation coefficient of the standardized reference samples was 0.91 ( Supplementary Figure S1B). The slight difference in Pearson correlation coefficients between real samples and standardized reference samples is attributed to individual variations between mice. Nonetheless, this evaluation confirms the consistency and reliability of our experimental results, allowing us to confidently proceed with further quantitative analyses.

### Properties of identified glycated peptides and proteins

**Figure 2:**
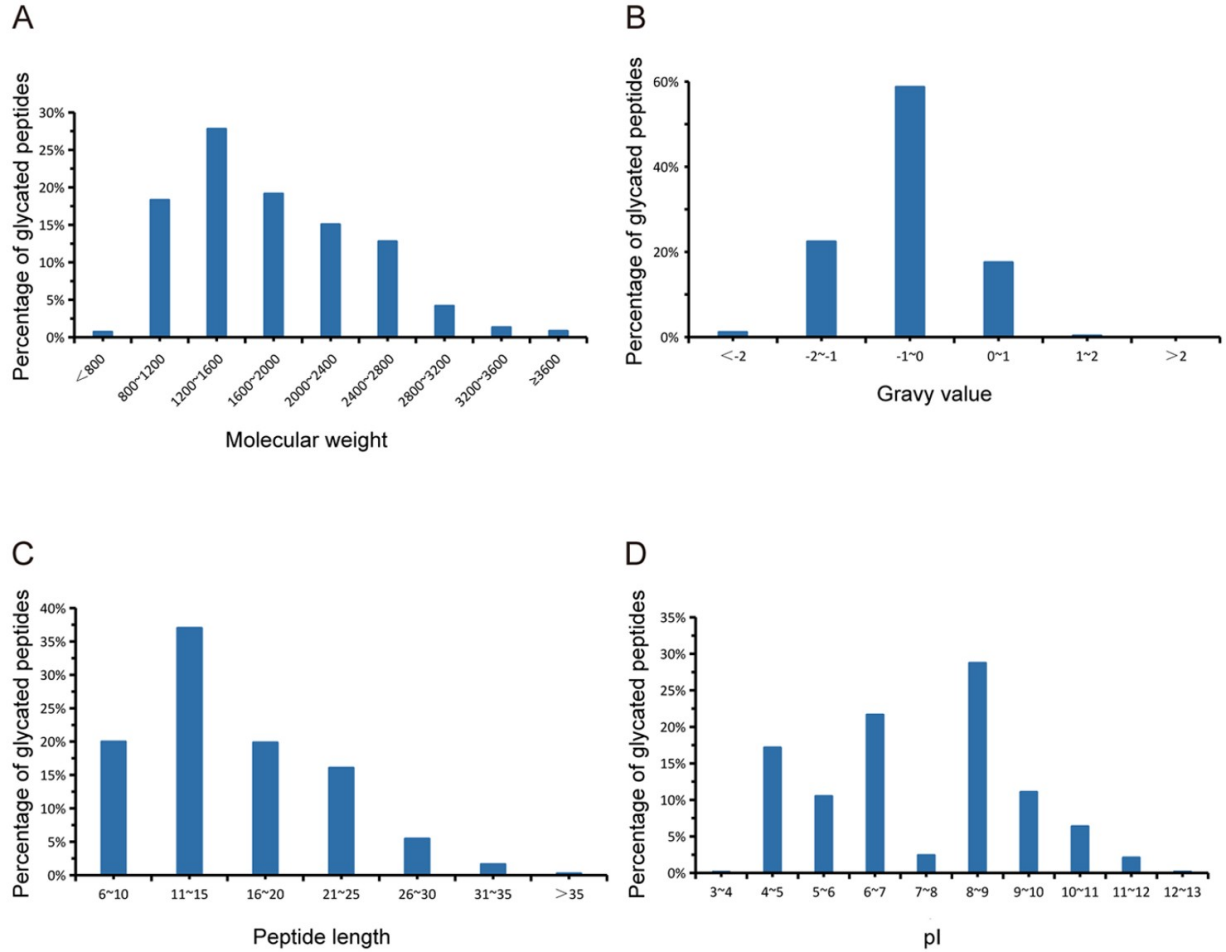
Figure 2 Properties of identified glycated peptides (A) Molecular weight. (B) Gravy value. (C) Peptide length. (D) pI (isoelectric point).

**Figure 3:**
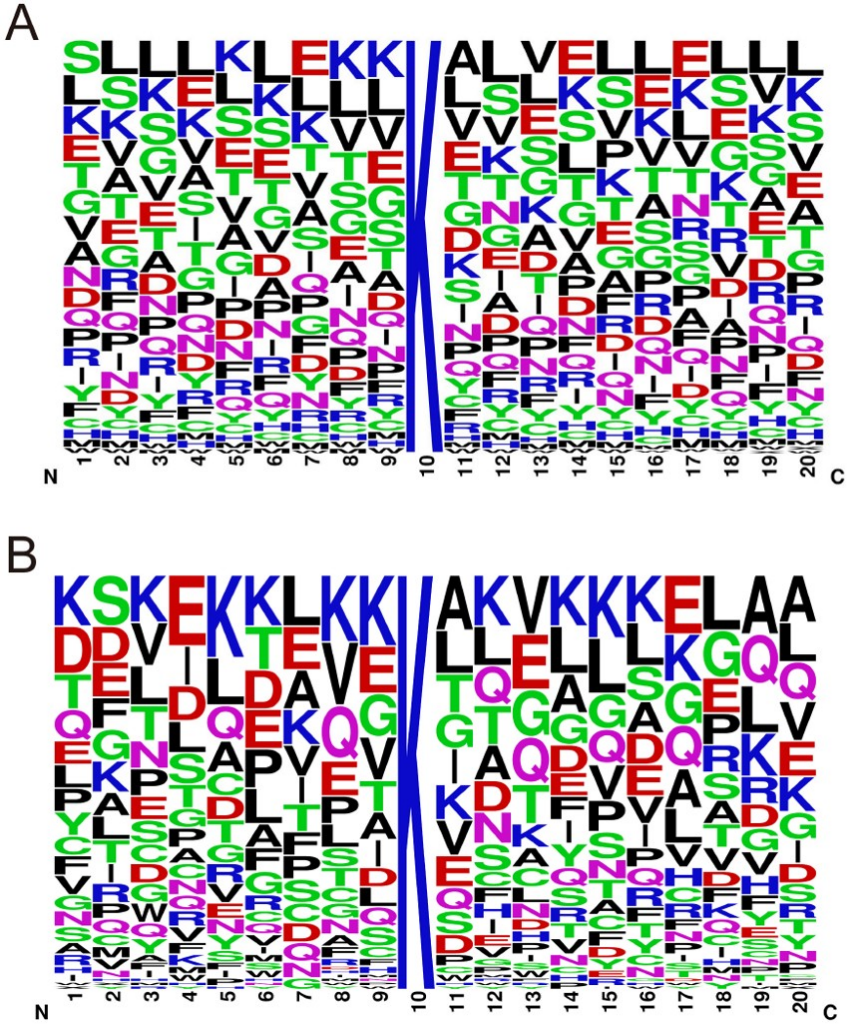
Figure 3 Weblogo analysis of amino sequence before and after identified glycated sites (A) Weblogo analysis of all the identified glycated peptides. (B) WebLogo analysis of glycated peptides with more than one identified glycation site.

We investigated the physicochemical properties of the identified glycated peptides ( Figure 2). Notably, 93% of the identified glycated peptides had a molecular weight ranging from 800 to 2800 Da, with 28% concentrated between 1200 and 1600 Da. The gravy value distribution of the identified glycated peptides was predominantly within the range of –2 to 1. In terms of peptide length, 37% of the identified glycated peptides consisted of 11 to 15 amino acid residues. Additionally, 49% of the identified glycated peptides had an isoelectric point lower than 7, while 51% had an isoelectric point higher than 7. Concerning the number of glycation sites, the majority (2848 out of 2959) had a single glycated site, 104 had two sites, and only six had three sites. Further analysis of the amino acid sequence before and after the identified glycated sites with Weblogo analysis ( Figure 3) showed no preferential residue at any specific position, indicating the absence of specific sequence motifs for glycated peptides with one or more glycated sites.

**Figure 4:**
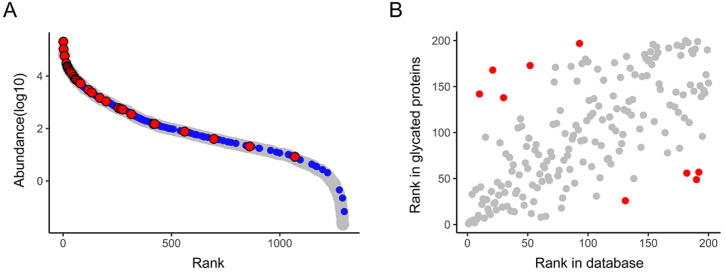
Figure 4 Distribution of serum early glycated protein abundance (A) Dynamic coverage of all identified glycated proteins by plotting the abundance distribution of the identified glycated proteins against the reported mouse plasma protein abundance distribution. The X-axis represents the rank of mouse plasma proteins based on the reported abundance distribution. The Y-axis represents log10 of glycated protein abundance. All the identified glycated proteins are shown in blue, while upregulated glycated proteins from 48 w to 80 w are shown in red. (B) The correlation of the abundance rank of identified glycated proteins with that of mouse plasma proteins in the database. The X-axis is the rank of the identified proteins in the database, and the Y-axis is the rank of early-glycated proteins we identified. Proteins with significant changes in rank are shown in red.

To assess the dynamic coverage of the identified glycated proteins, we compared the abundance distribution of these proteins to the reported mouse plasma protein abundance distribution ( Figure 4 A) [24]. The abundance distribution of the identified glycated proteins effectively covered the full dynamic range of mouse plasma proteins, indicating that both high-abundance and low-abundance proteins in mouse plasma can be glycated. The results demonstrated that our method is sensitive for the identification of low-abundance proteins, and both high-abundance proteins and low-abundance proteins have the opportunity to be glycated. Interestingly, among the upregulated glycated proteins from 48 w to 80 w, high-abundance proteins were predominant. This observation suggests that high-abundance proteins may be more exposed to reducing sugars and more susceptible to glycation during aging.

Figure 4A shows that the upregulated glycated proteins from 48 w to 80 w were mainly high-abundance proteins. Figure 4B further reveals that the abundance of identified serum glycated proteins correlates well with that of mouse plasma proteins, with a correlation coefficient of 0.694. However, we identified 9 proteins with markedly different glycated levels and protein abundance ranks (ranking difference of more than 100), suggesting that not only protein abundance but also protein structure could impact the level of glycation modification ( Supplementary Table S1).

### Glycated proteome changes in different age groups

**Figure 5:**
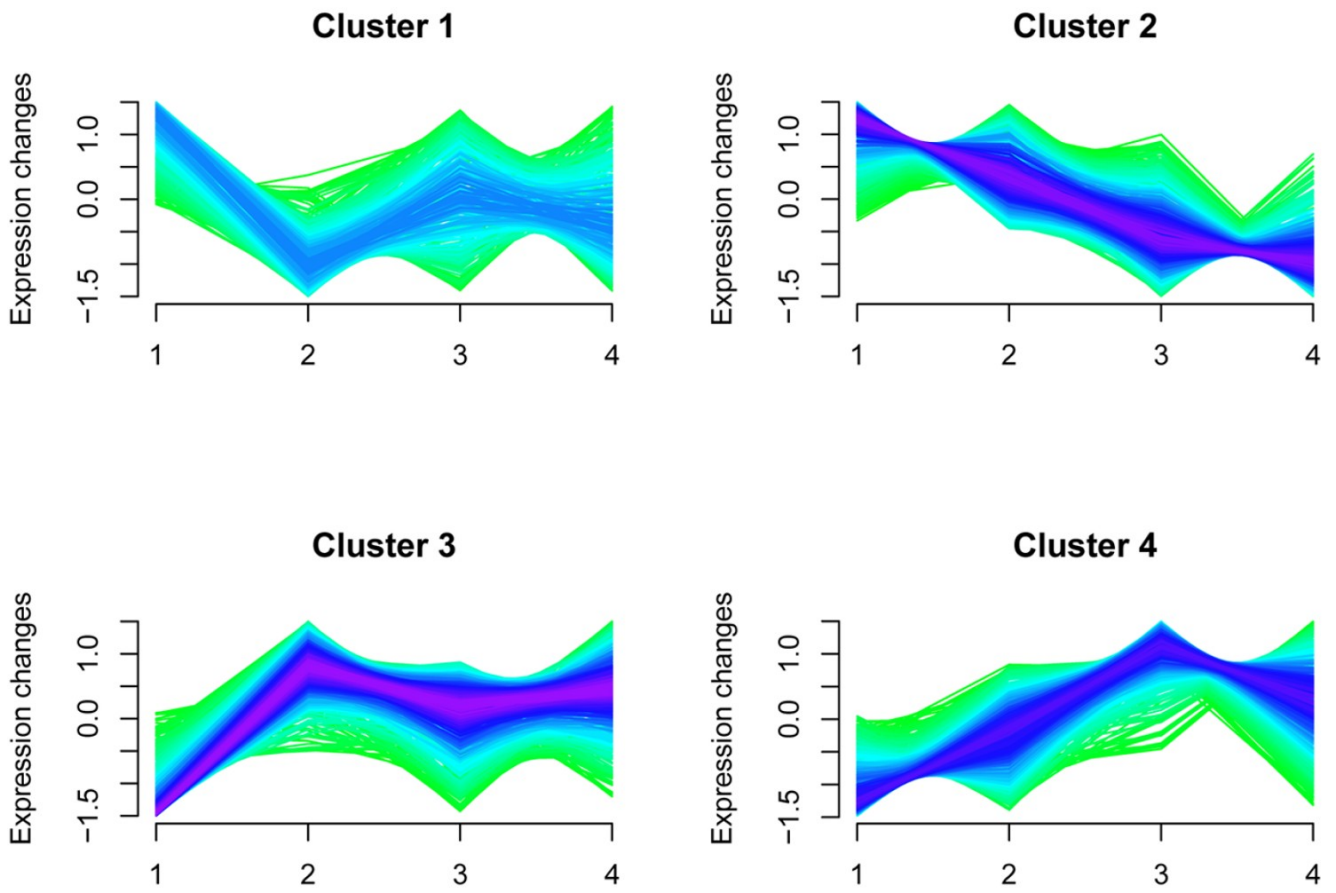
Figure 5 Identification of specific clusters of glycated peptides A total of 1957 glycated peptides were clustered using mFuzz into 4 significant discrete clusters to illustrate the relative expression changes in the proteomics data.

To understand the temporal patterns of glycated proteins in different age groups, we evaluated these patterns by mFuzz analysis ( Figure 5). We identified 4 distinct clusters representing different early-glycated protein expression kinetics. Cluster 2 displayed downregulated glycated peptides, cluster 3 showed glycated peptides with an initial upregulation followed by stability, and clusters 1 and 4 represented glycated peptides with bimodal expression patterns.

**Figure 6:**
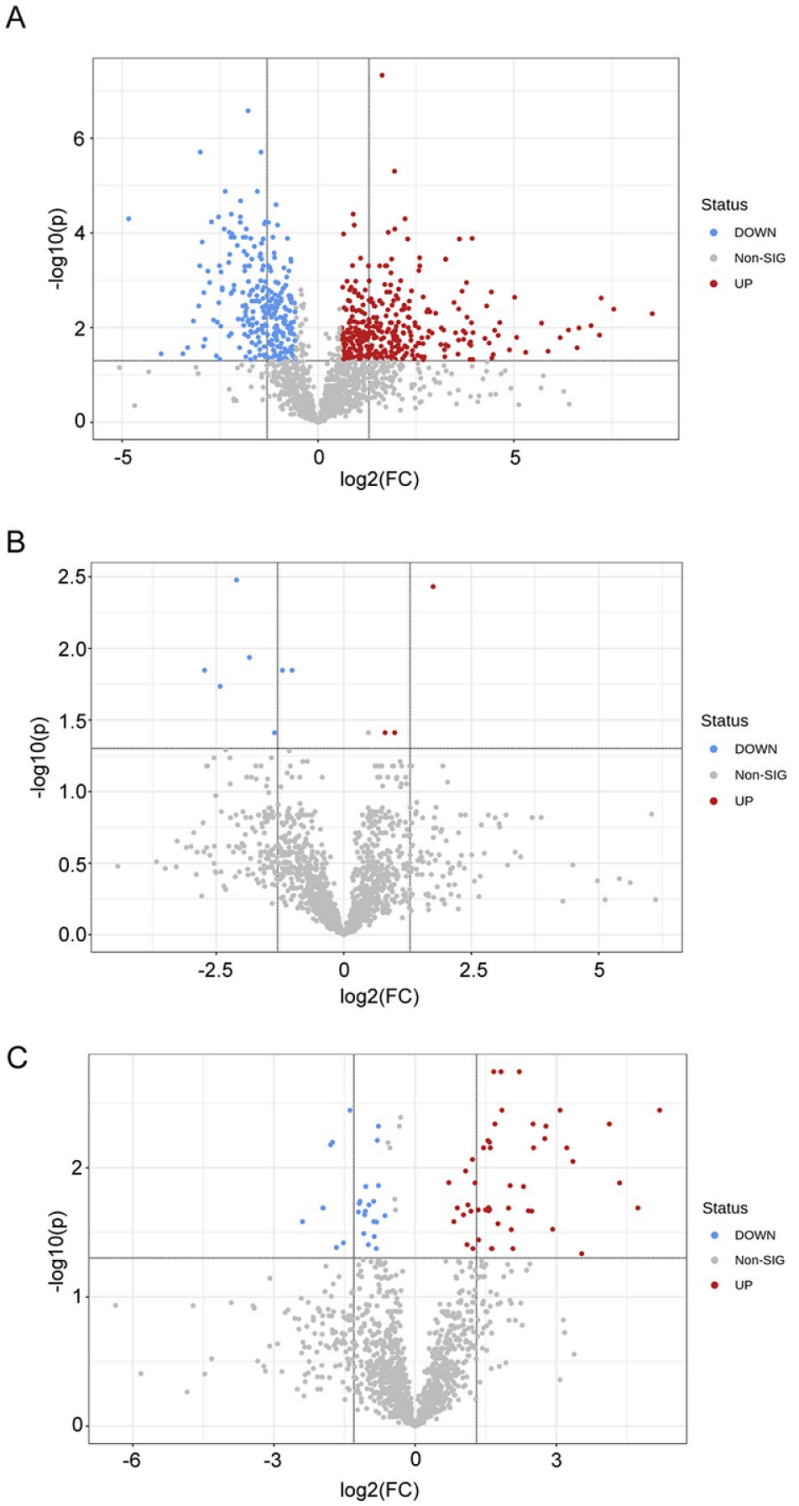
Figure 6 Differentially expressed glycated proteomes in different age groups of mice Volcano plots comparing 3 pairs of age groups (A: 10 w & 16 w; B: 16 w & 48 w; C: 48 w & 80 w). Glycated peptides with FC beyond 1.5 or below 0.67 with adjusted P values lower than 0.05 were considered significantly differentially expressed. Significantly upregulated features are in red, and significantly downregulated features are in blue. The remaining features are in grey.

Subsequently, we compared the serum early-stage glycated proteome between different age groups using MetaboAnalyst ( Figure 6). We identified 347 significantly upregulated and 257 significantly downregulated glycated peptides when comparing 10 w and 16 w mouse serum samples. The transition from 16 w to 48 w exhibited minor changes, with only 3 significantly upregulated and 7 significantly downregulated glycated peptides. Between the 48 w and 80 w age groups, we observed 49 significantly upregulated and 26 significantly downregulated glycated peptides. Moreover, the number of significantly upregulated glycated proteins was 103, 3 and 35 in each two-group comparison. Interestingly, 10 glycated proteins (or protein groups) were found to be significantly upregulated only between 48 w and 80 w. Among these proteins, lecithin-cholesterol acyltransferase, apolipoprotein A-II, and others showed significant upregulation only in the 48 w to 80 w transition ( Supplementary Table S2).

**Figure 7:**
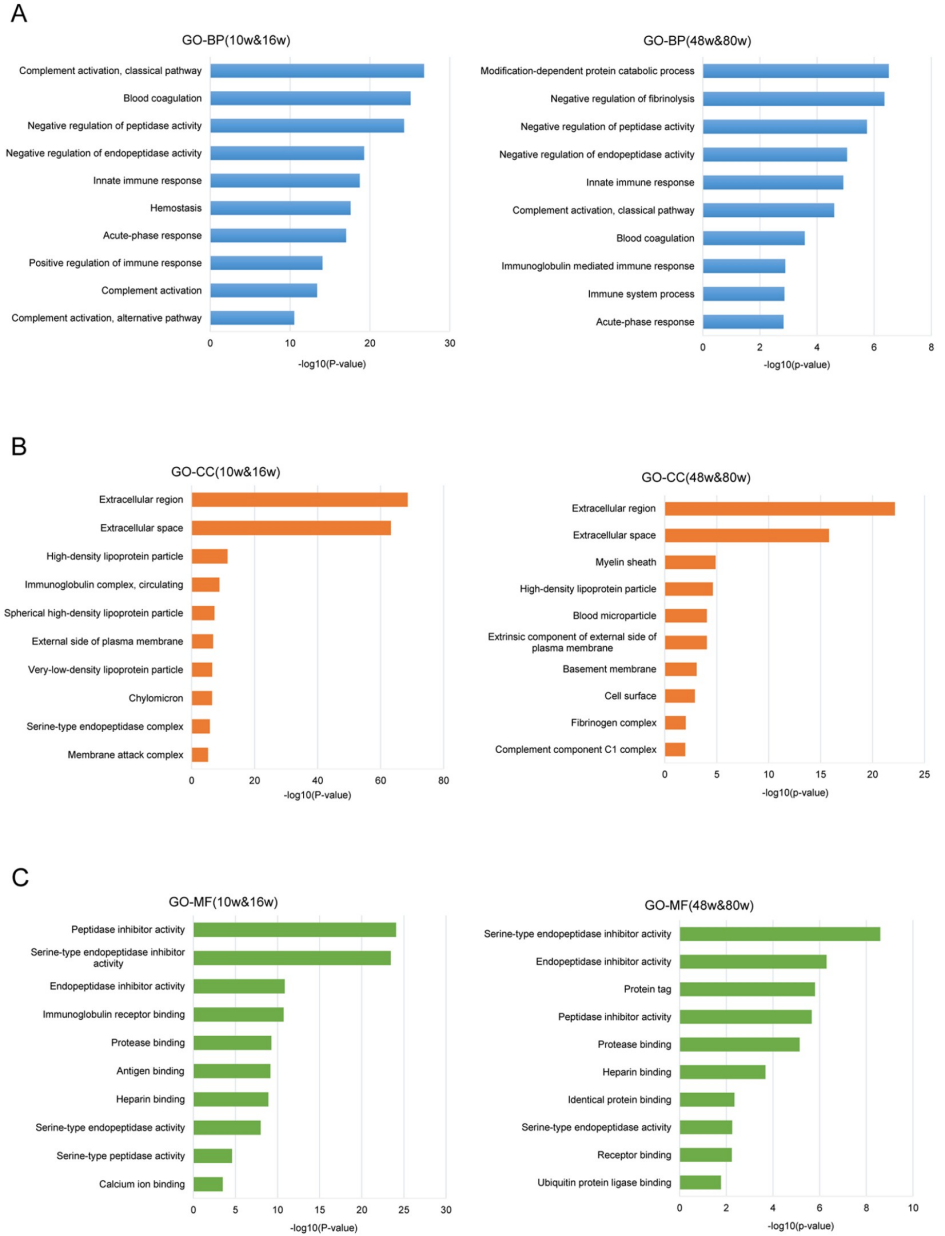
Figure 7 Gene ontology (GO) enrichment analysis of significantly upregulated proteins in two age stages (age stage of 10 w & 16 w and age stage of 48 w & 80 w) (A) GO biological process. (B) Cellular component. (C) Molecular function enrichment analysis.

To gain insights into the cellular functions and biological processes affected during aging, we performed gene ontology (GO) enrichment analysis of the identified upregulated proteins, both in the younger age stage of 10 w versus 16 w and the older age stage of 48 w versus 80 w ( Figure 7). We found common biological pathways and molecular functions enriched in both younger and older stages, indicating that proteins related to these pathways and functions are more likely to undergo glycation modification changes. However, unique pathways and molecular functions were also enriched in the older age stage, such as the pathway of modification-dependent protein catabolic process ( Figure 7A) and the function of receptor binding and ubiquitin protein ligase binding ( Figure 7C). These findings suggest potential targets for aging and aging-related disease biomarkers and intervention approaches. Moreover, the analysis showed that both extracellular region and space were the top two enriched cellular compartments for both stages ( Figure 7B), consistent with the serum sample source and validating the reliability of the data.

A total of 112 signaling pathways related to aging have been enriched based on numerous proteomic studies performed in different matrices and species [6]. To investigate whether early-glycated proteins are similarly enriched in these pathways, we found that upregulated proteins in the 48 w and 80 w age groups were significantly associated with GO categories related to modification-dependent protein catabolic processes, negative regulation of peptidase activity, innate immune response, complement activation, negative regulation of fibrinolysis, and blood coagulation. Among these pathways, innate immune response, complement activation, and blood coagulation align with previous proteomic research [6], while others represent newly discovered pathways related to aging. These findings provide further insights into the role of glycation in age-related processes.

Overall, our study reveals comprehensive and quantitative profiling of serum-glycated peptides in different age groups of mice. We identified age-related changes in the glycated proteome and observed distinct temporal patterns of glycated proteins. Our findings shed light on potential targets for aging biomarkers and intervention strategies and contribute to a better understanding of the role of glycation in age-related processes.

## Discussion

Our study represents a significant advancement in the field of glycated proteome analysis, as it integrates filter-aided sample preparation technology, selective enrichment of the glycated proteome, and a label-free quantitative method to comprehensively explore early glycated proteome changes during aging in mouse serum. We successfully identified 2959 endogenous glycated peptides from 296 serum proteins, making this dataset the largest collection of endogenous glycated proteins identified in the context of aging to date.

Additionally, we obtained valuable information about the properties of glycated peptides and proteins, including molecular weight, gravy value, peptide length, isoelectric point, glycation number, position preference, and abundance distribution. These findings provide comprehensive insights into the characteristics of endogenous glycation modification and offer potential targets for interventions aimed at reversing early glycation.

Previous studies on serum early-glycated proteins predominantly focused on individual high-abundance proteins, such as glycated hemoglobin, glycated albumin, and glycated transferrin [ 15 , 25, 26]. In contrast, our advanced proteomics platform allows for the detection of both highly abundant and low-abundance glycated proteins. The broad dynamic range of the detected glycated proteins validates the superior performance of our glycated proteomics method for complex biological sample analysis, where high coverage results are required across a wide dynamic range of protein concentrations. We successfully identified age-related early glycated proteins, including glycated hemoglobin, glycated albumin, and glycated transferrin, confirming the reliability of our method [ 15, 25, 26]. Furthermore, we discovered novel early-glycated proteins, indicating that the occurrence of protein glycation may be related not only to protein concentration but also to protein structure.

The accumulation of advanced glycation end products (AGEs) is a known hallmark of aging and metabolic diseases [27]. However, few studies have investigated the relationship between early glycated proteins and aging, partially due to the lack of high-sensitivity and high-throughput glycated proteome analysis techniques. Nevertheless, it is essential to recognize that the formation of early glycated proteins plays a crucial role in promoting the accumulation of AGEs [28]. In our study, we identified 10 glycated proteins (or protein group), including LCAT, Apo A-II, ubiquitin C (UBC), and complement component 1, r subcomponent A (C1rA), which increased exclusively in the old age group when comparing serum glycated proteomes from mice at four different age time points. These glycated proteins may be closely related to aging and could potentially serve as physiological age biomarkers.

Investigating the functions and mechanisms of these glycated proteins specifically changed in the aged group may provide new insights into the study of aging and aging-related diseases. For instance, LCAT is an enzyme mainly synthesized by the liver and secreted in plasma, where it catalyzes the formation of cholesterol esters from free cholesterol in nascent HDL to facilitate HDL particle maturation [29]. Previous studies have linked LCAT activity to atherosclerotic cardiovascular disease risk [30], and altered function due to gene mutations has been associated with atherosclerotic pathology [31]. Although glycation modification of LCAT has not been reported before, other modifications, such as glycosylation, have been found to influence LCAT function and are associated with cardiovascular disease, an age-related condition [ 32, 33]. Glycation and glycosylation are both modified in the LCAT protein. The difference is that the former is non-enzymatic and the latter is enzymatic [34]. We speculate that increased glycation modification in older mice may potentially affect LCAT function, impacting cholesterol metabolism and levels of important lipoproteins such as apolipoprotein E. Therefore, further investigation into the correlation between LCAT glycation and aging, aging-related diseases, and its potential role as a biomarker and intervention target is warranted. Similarly, other newly discovered aging-related early glycation proteins, such as polyubiquitin-C, present new molecular clues for the study of aging and aging-related diseases. Understanding the function and significance of these proteins in the context of glycation and aging may open up new avenues for biomarker discovery and interventions in related fields.

In conclusion, our study offers a comprehensive analysis of serum early-glycated proteome changes during aging spanning from young to old in mice, highlighting the significance of integrating advanced proteomics techniques for glycated proteome analysis. We have identified numerous glycated proteins and provided valuable insights into their properties, functional relevance, and potential implications in aging and aging-related diseases. To our knowledge, our findings represent the largest dataset of aging-related endogenous early-glycated proteome information to date. Notably, we identified 10 glycated proteins (groups) that are uniquely and significantly upregulated in the old age group, including LCAT and Apo A-II. Additionally, we uncovered new biological pathways, such as modification-dependent protein catabolic processes and negative regulation of peptidase activity, which are related to aging through early protein glycation. Our findings pave the way for future research on the role of glycation in aging processes and may contribute to the development of novel biomarkers and therapeutic strategies for aging-related conditions.

## Supporting information

353supplementary_information

## Supplementary Data

Supplementary data is available at *Acta Biochimica et Biophysica Sinica* online.
