# Supplementary material for: Discovery of age-related early-stage glycated proteins based on deep quantitative serum glycated proteome analysis: Age-related changes in the early-glycated proteome
Source: Acta Biochim Biophys Sin (Shanghai). 2023 Aug 31;55(10):1659–67. doi: 10.3724/abbs.2023222 (PMC10577472; doi:10.3724/abbs.2023222)
Supplement: 353supplementary_information [file 353supplementary_information.pdf]

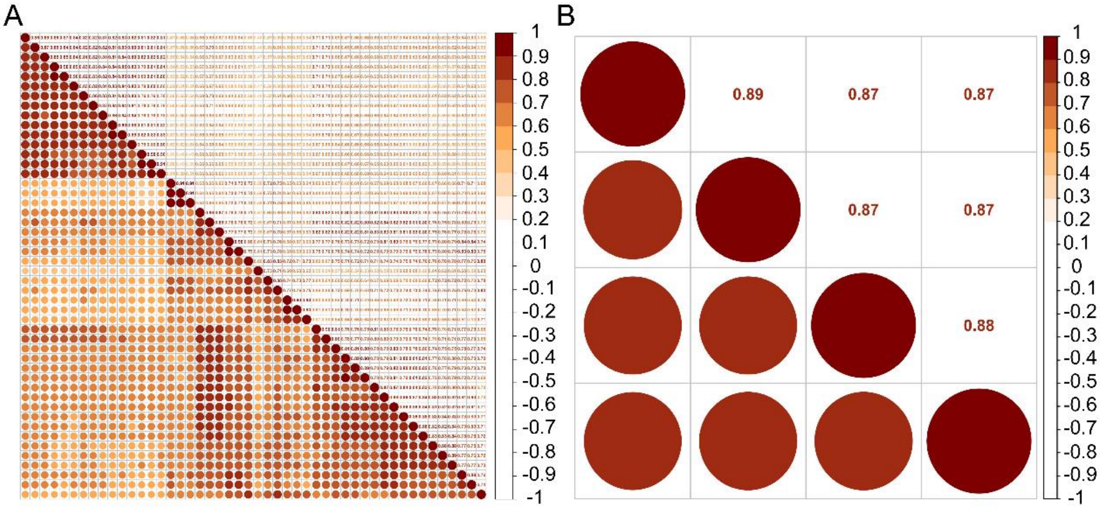

Supplementary Figure S1. Stability evaluation (A) Pearson correlation coefficients for glycated peptide profiling of all serum samples. (B) Overview of quality control for the LC-MS platform using digested HeLa protein as the reference sample (four runs).

Supplementary Table S1. Proteins with markedly different glycated levels and protein abundance ranks (ranking difference of more than 100)

Protein accession	Rank in database	Rank in glycated proteins
Q03734|SPA3M_MOUSE	10	142
Q8K0E8|FIBB_MOUSE	21	168
E9PV24|FIBA_MOUSE	30	138
Q9DBB9|CPN2_MOUSE	52	173
P11588|MUP1_MOUSE	93	197
P06684|CO5_MOUSE	131	26
Q9Z126|PLF4_MOUSE	182	56
P63101|1433Z_MOUSE	190	49
P35441|TSP1_MOUSE	192	57

Supplementary Table S2. Glycated proteins (or protein groups) significantly upregulated only between 48 w and 80 w

Protein accession	Glycated peptide	FC	*P*.ajusted
Q9DBB9|CPN2_MOUSE	AFSGSPNLTK(+164.07)VVFLN(+0.98)TQVR	3.54	1.80×10^{-3}
P16301|LCAT_MOUSE	LAPHQQDEYYKK(+164.07)	3.18	1.80×10^{-3}
P09813|APOA2_MOUSE	DLMEK(+164.07)AK(+164.07)TSEIQSQAK	17.46	4.58×10^{-3}
O70362|PHLD_MOUSE	N(+0.98)HTLSGSK(+164.07)VQK	2.41	1.31×10^{-2}
Q8CFG9|C1RB_MOUSE; Q8CG16|C1RA_MOUSE	VLNYVDWIKK(+164.07)	4.05	1.37×10^{-2}
P0CG50|UBC_MOUSE; P62983|RS27A_MOUSE; P62984|RL40_MOUSE; P0CG49|UBB_MOUSE	LIFAGK(+164.07)QLEDGR	5.30	2.15×10^{-2}
Q9ESB3|HRG_MOUSE	LGC(+57.02)PPPPEGK(+164.07)DNSDRPR	1.77	2.61×10^{-2}
P61939|THBG_MOUSE	INSYVEK(+164.07)QTK	2.55	3.61×10^{-2}
Q9D3H2|OBP1A_MOUSE	FAELAEEK(+164.07)GIPAGNIR	2.15	3.93×10^{-2}
P82198|BGH3_MOUSE	STVISYEC(+57.02)C(+57.02)PGYEK(+164.07)VPGEK	2.34	4.22×10^{-2}
